# Primary cardiac myxofibrosarcoma of the left atrium and pericardium: a case report

**DOI:** 10.1186/s13019-023-02441-9

**Published:** 2023-11-16

**Authors:** Ryota Azuma, Kazuyoshi Sato, Hiroki Sunadoi, Yasushi Ishii, Utano Tomaru, Masatoshi Motohashi

**Affiliations:** 1 Department of Cardiovascular Surgery, Hakodate Central General Hospital, 33-2 Honcho, Hakodate, Hokkaido 040-8585 Japan; 2 Department of Pathology, Faculty of Medicine, Graduate School of Medicine, Hokkaido University, Sapporo, Japan

**Keywords:** Primary cardiac myxofibrosarcoma, Left atrium, Pericardium

## Abstract

**Background:**

Primary cardiac myxofibrosarcoma is rare and commonly occurs in the left atrium. Myxofibrosarcoma is aggressive and has a high mortality rate due to its high rate of recurrence. Complete surgical resection is considered important; however, effective treatment options have not been established.

**Case presentation:**

We report the case of a 75-year-old woman who developed a myxofibrosarcoma spreading to the left atrium and pericardium. We performed surgical resection of the tumor to prevent sudden death due to mitral valve obstruction or cerebral infarction due to embolism of the scattered mass. However, we were unable to complete the resection of the tumors. The patient developed brain metastasis 2 months after surgery and eventually died due to brain hemorrhage 3 months after surgery.

**Conclusions:**

In this report, we described a rare case of primary cardiac myxofibrosarcoma located not only in the left atrium but also in the pericardium. Considering preoperative laboratory findings, surgical and adjuvant therapy, and the patient’s wishes are important for the best therapeutic course for an individual.

## Background

Myxofibrosarcoma is a malignant tumor of mesenchymal origin that most commonly appears in the extremities of older patients [1]. Primary cardiac myxofibrosarcoma is rare and has only been reported in isolated case reports [2]. Primary cardiac myxofibrosarcoma commonly arises in the left atrium (LA) [2]. This type of tumor has a high mortality rate because of local invasion or high rate of recurrence [3]. Complete surgical resection is considered important to improve the prognosis; however, effective treatment options have not been established [4]. In this report, we describe a rare case of primary cardiac myxofibrosarcoma that arose in both the LA and pericardium.

## Case presentation

**Fig. 1:**
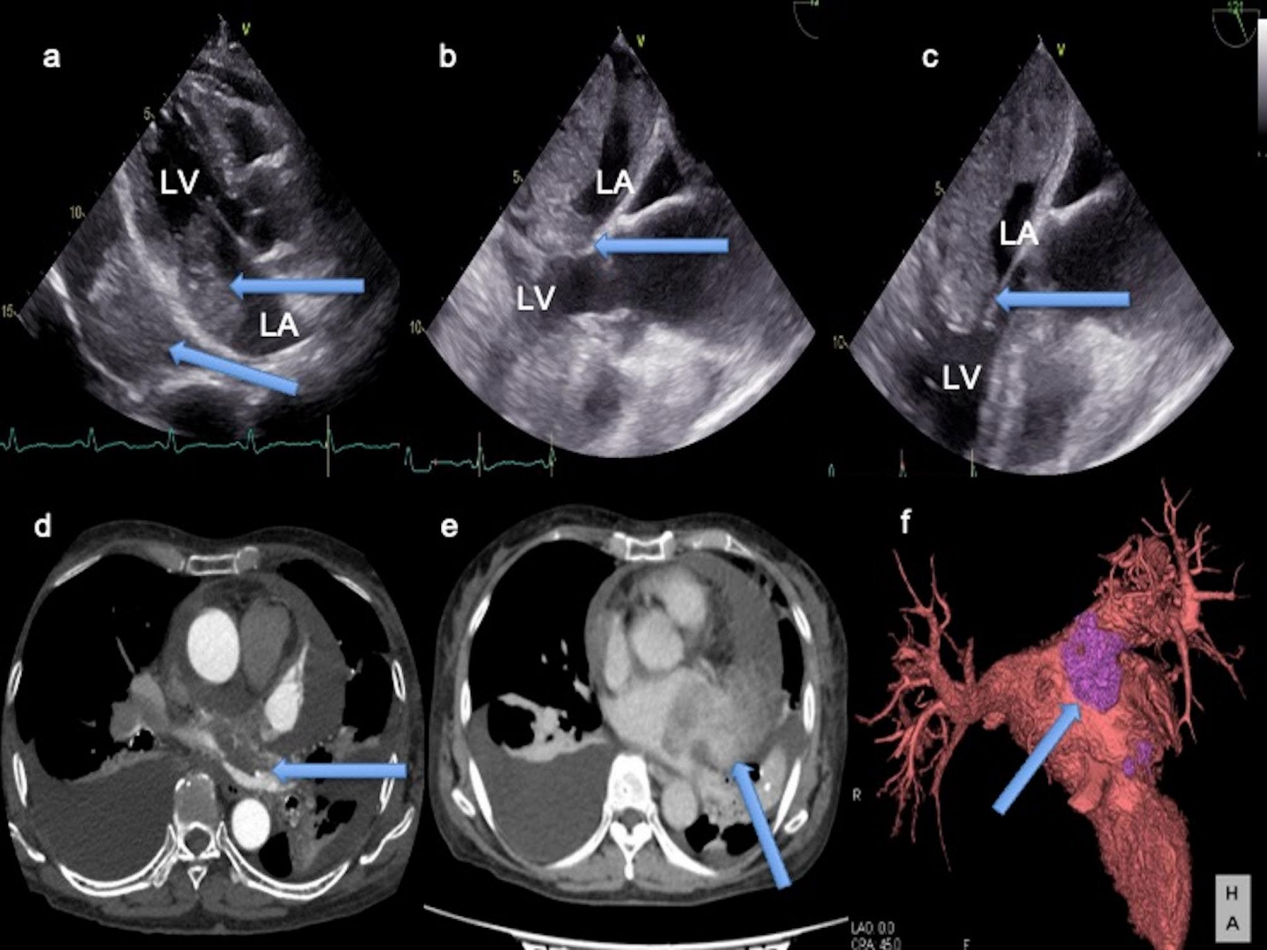
Preoperative transthoracic echocardiogram (TTE), transesophageal echocardiogram (TEE), and computed tomography (CT) images **(a)** TTE revealing a movable mass in the left atrium (LA) and another mass in the pericardium. **(b)** TEE showing the tumor in the LA during the systolic phase. **(c)** LA tumor prolapsed across the mitral valve during the diastolic phase. **(d, f)** CT and three-dimensional CT revealing a mass in the LA extending to the left upper PV, **(e)** Both masses were heterogeneously different. LV: left ventricle

A 75-year-old woman was brought to our hospital with a 2-week history of progressive dyspnea and edema. Chest radiography revealed bilateral pleural effusions. A transthoracic echocardiogram (TTE) revealed a movable mass (45 × 25 mm) occupying the LA and another mass (68 × 43 mm) in the pericardium (Fig. 1a). TTE also showed pericardial fluid. A transesophageal echocardiogram (TEE) showed that both masses originated from the posterior LA wall and seemed to be connected. The mass in the LA prolapsed across the mitral valve, without incarceration (Fig. 1b) and extended to the left upper pulmonary vein (PV). Contrast-enhanced computed tomography (CECT) revealed that the lobular mass in the LA extended from the posterior LA wall to the left upper PV (Fig. 1c). The masses in the LA and pericardium were heterogeneously different (Fig. 1d). Cardiac magnetic resonance imaging (MRI) showed that both masses had a high intensity relative to the heart muscle on T1- and T2-weighted sequences. The enhancement sequences showed multiple low-intensity zones within both the masses. CECT and brain MRI revealed no metastatic foci. The CT and MRI findings suggested that the masses were malignant, but we could not completely rule out the possibility that they were benign, partly due to being mobile. If the masses were malignant, completely resection would be difficult since the LA mass extended to the left upper PV and the prognosis was of only a few months. Considering that the patient was older, surgical treatment would not be recommend. However, if the masses were benign, surgical resection would be indicated to prevent the high potential risk of sudden death due to obstruction of the mitral valve or cerebral infarction due to embolism of the scattered mass. In addition, we could get a definitive diagnosis. Eventually, the patient wished to perform surgery to prevent sudden death and cerebral infarction, so we decided to perform surgical resection of both masses urgently.

**Fig. 2:**
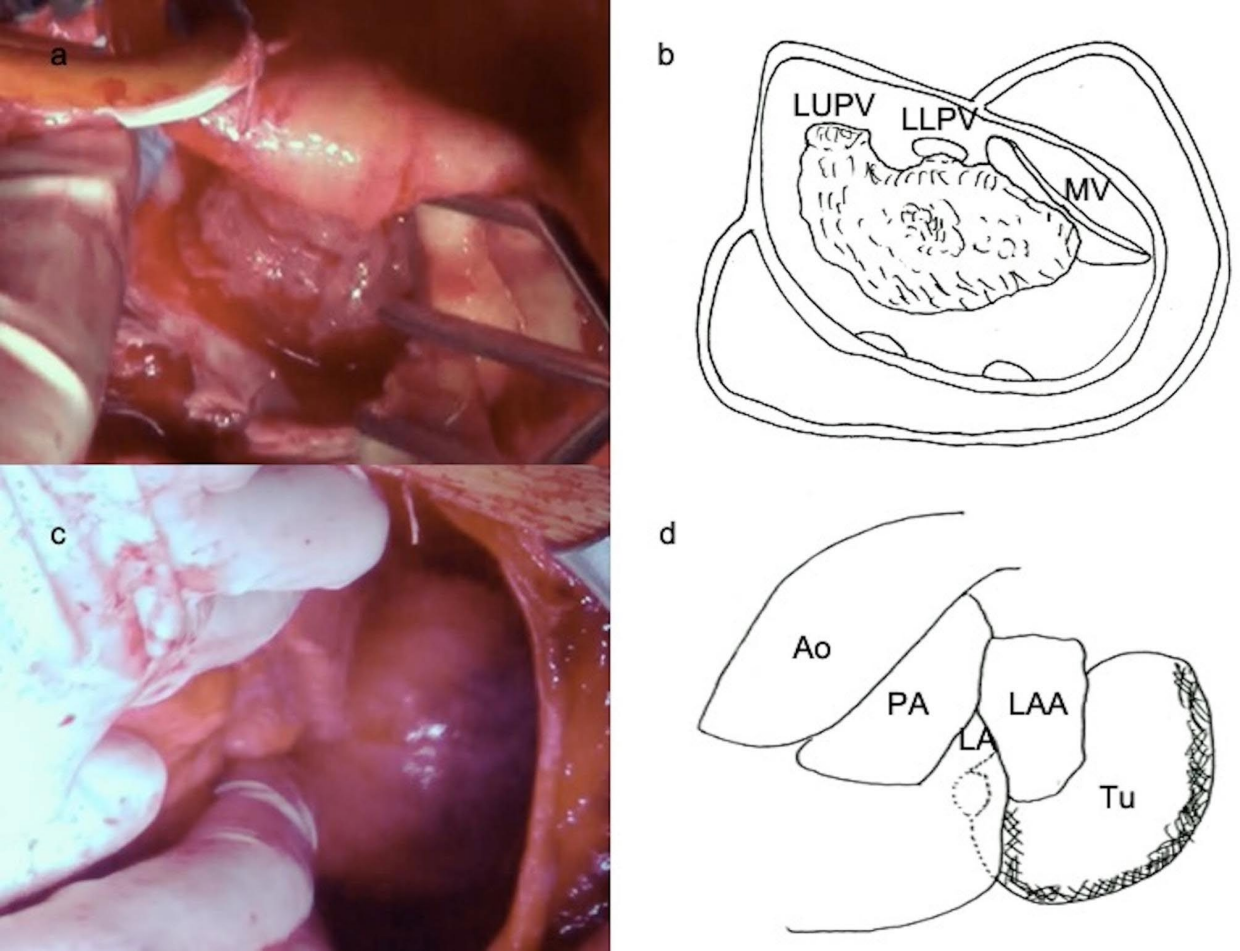
Surgery pictures and schemas **(a)** The tumor in the left atrium (LA) appears to be a myxoma. **(b)** LA tumor is widely attached to the posterior LA wall and extends to the posterior mitral leaflet and left upper pulmonary vein (LUPV). **(c)** The pericardial tumor is smooth. (d) The tumor in the pericardium is connected to the posterior LA wall. LLPV: left lower pulmonary vein; MV: mitral valve; Ao: aorta; PA: pulmonary artery; LAA: left atrial appendage; Tu: tumor

After the median sternotomy, the LA was approached through the superior septal. In total, 600 mL of bloody pericardial fluid was collected. Macroscopically, the tumor in the LA appeared to be a myxoma (Fig. 2a). The tumor was fragile and widely attached to the posterior LA wall and extended to the posterior mitral leaflet (PML) and the left upper PV (Fig. 2b). The PML was not infiltrated, but the left upper PV was infiltrated and obstructed by the tumor. The pericardial tumor was connected to the LA tumor at the posterior LA wall and macroscopically, it was smooth (Fig. 2c). The pericardial tumor was completely resected from the LA wall. The tumor in the LA was grossly resected along with the LA posterior wall; however, we could not remove the tumor in the PV. Finally, we reconstructed the posterior LA wall defect (20 × 30 mm) using a bovine pericardial patch.

**Fig. 3:**
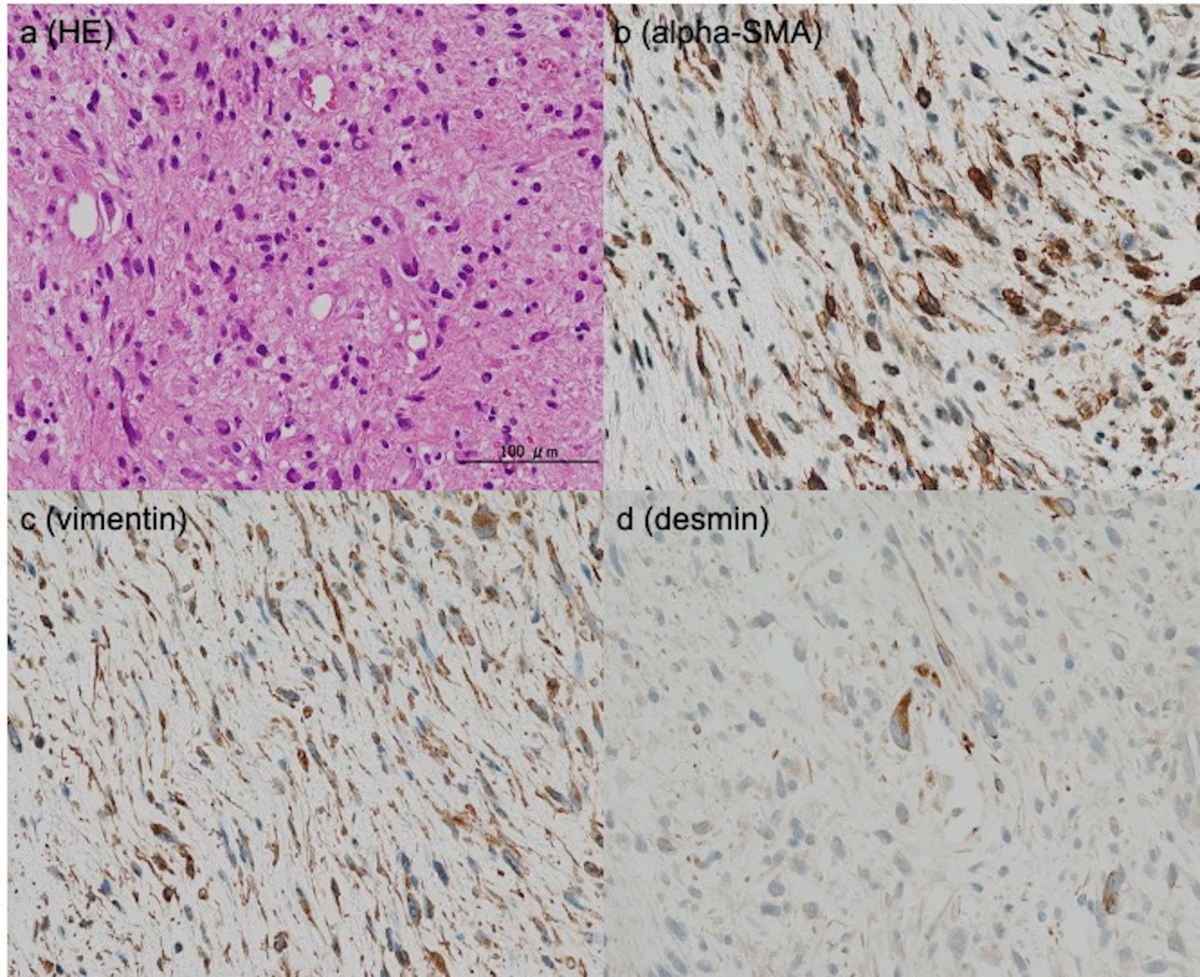
Histological images **(a)** An examination with hematoxylin and eosin staining technique showed spindle shaped and pleomorphic cells with hemorrhage and necrosis. Bizarre, multinucleated giant cells, and cells with irregular-shaped nuclei were often noted. (**b**, **c**, and **d**) Immunohistochemical tests showed tumor cells were positive for alpha-SMA and vimentin, and rarely positive for desmin

Histologically, the tumor was composed of spindle shaped and pleomorphic cells with areas of hemorrhage and necrosis. Bizarre, multinucleated giant cells, and cells with irregular-shaped nuclei were often noted. Immunohistochemical tests revealed that the tumor cells were positive for vimentin and alpha-SMA, partially positive for CD34 and HHF-35, and rarely positive for desmin (Fig. 3). Therefore, the patient was finally diagnosed with myxofibrosarcoma.

The patient had an uncomplicated postoperative course. Brain and whole-body CT showed no recurrence or metastatic regions at 1 month after surgery. We considered postoperative chemotherapy or radiotherapy was necessary as soon as possible because the resection of the PV tumor was incomplete; however, the patient did not wish to receive adjuvant therapy. At 2 months after surgery, a metastatic region was found on the brain CT. At 3 months postoperatively, the patient complained of a sudden headache and nausea. Brain CT showed a brain hemorrhage in the metastatic region, and the patient died.

## Discussion and conclusions

Primary cardiac tumors are very rare. The incidence is between 0.0017% and 0.03% in autopsy series. Most primary cardiac tumors are benign, but approximately 25% are malignant [5]. Among primary malignant cardiac neoplasms, approximately 80% are sarcomas. Angiosarcomas are the most common, followed by rhabdomyosarcomas, malignant mesotheliomas, and fibrosarcomas [6].

Myxofibrosarcoma is a malignant tumor of mesenchymal origin that most commonly occurs in the extremities of older patients and a primary myxofibrosarcoma rarely arises in the heart [4]. The clinical presentation often varies. Similar to other benign or malignant cardiac tumors, cardiac myxofibrosarcoma presents with symptoms such as dyspnea, chest pain, edema, and syncope. The LA is the most common location of the tumor, followed by the LA + PV, right heart system, and left ventricle [2]. We searched the number of cases of primary cardiac myxofibrosarcoma that have been reported using the Web of Science database, with keywords, “myxofibrosarcoma” and “cardiac.” There were 38 case reports of primary cardiac myxofibrosarcoma, but only one case was reported of a tumor located in the pericardium [7]. Thus, to the best of our knowledge, this is the first case report of primary cardiac myxofibrosarcoma occurring in both the LA and pericardium.

Distinguishing myxoma from myxofibrosarcoma is important because myxoma should be promptly resected to prevent sudden death or embolic event [8]. Currently, cardiac myxofibrosarcoma is difficult to differentiate from myxomas because both tumors are mainly attached to the LA wall [9]. In addition, because the histological types of cardiac tumors vary, characterization of cardiac tumors using imaging techniques has been challenging [9]. In our case, the echocardiogram and CT showed that the tumors were in the LA wall. However, the masses existed in both the LA and pericardium, and the LA mass was extended to the PV. Therefore, the tumors were not considered to be myxoma. MRI showed that both masses had high intensity on T2-weighted sequences and multiple low-intensity zones within the masses on enhancement sequences. John P et al. reported that undifferentiated sarcomas showed high intensity on T2-weighted sequences and heterogeneity on enhancement sequences [8]. Therefore, we assumed that the masses were sarcomas. However, we could not completely rule out the possibility that the tumor was benign, partly because it was mobile. In our case, image modalities showed pericardial fluid. We did not perform pericardiocentesis because hemodynamics was stable, and we planned to attempt tumor resection for preventing sudden death and embolic event regardless of whether the tumors were benign or malignant. Although gaining a definitive diagnosis from only pericardial fluid is difficult, we could gain additional findings which contribute to distinguishing malignancy from the fluid properties or cytological examination. The examination of pericardial fluid would be valuable to evaluate the prognosis.

The median survival time of primary cardiac myxofibrosarcoma patients treated with tumor resection is only 14 months [2]. Although complete surgical resection is important for prolonged survival, it is difficult, and only occurs in one-third of cases [10]. In our case, the masses were expected to be difficult to resect completely. The masses existed not only intracavitary, but also in the pericardium, and the patient was older. Considering these points, there is a treatment option: First, we resect only the pericardial tumor for intraoperative diagnosis. Then, if the tumor is suspected to be benign, complete resection is performed under cardiopulmonary bypass. For this option, if the tumor is suspected to be malignant, we could reduce surgical stress by not resecting the LA mass and gain a definitive diagnosis from the pericardial mass; however, we could not prevent sudden death. We chose the option of resecting both masses under cardiac standstill because we wanted to avoid detaching the LA tumor by cardiac uphold or unexpected arrhythmia at the time of resecting the pericardial tumor. The reason for operating was to prevent sudden death or embolic event from incarceration of the LA mass. Consequently, although we could not complete the resection, we could prevent sudden death or embolic event, as the patient wished. Recently, it has been reported that cryoablation can cause tumor tissue necrosis and apoptosis. Moreover, these effects may prevent the progression of the primary tumor and distant metastasis [11]. Ujihara et al. reported that cryoablation is an effective procedure to resect the non-transmural atrial wall when tumors are resected incompletely [4]. In our case, we should have use cryoablation for the remaining PV tumor.

Myxofibrosarcoma is categorized as a low grade to high grade neoplasm. Low grade myxofibrosarcoma tends to metastasize locally, whereas high grade myxofibrosarcomas invade local and adjacent tissues and are associated with distant metastasis to the lung, bone, brain, and lymph nodes [10]. The local recurrence and distant metastasis rates were 42.9% and 19.0%, respectively [4]. Therefore, if the tumor is a high grade myxofibrosarcoma or cannot be completely removed, additional radiotherapy and chemotherapy should be considered. However, the rarity of this tumor is an obstacle in establishing effective chemotherapy regimens and radiotherapy doses. In our case, although we prevented sudden death or embolic event, the patient died after 3 months because of incomplete surgical resection and not performing early additional radiotherapy and/or chemotherapy.

In summary, we report a rare case of primary cardiac myxofibrosarcoma located not only in the left atrium but also in the pericardium. Distinguishing myxofibrosarcoma from myxoma or other malignant tumors is difficult preoperatively. To decide the best therapeutic course for the individual patient, considering in depth preoperative laboratory findings as many as possible, several options of surgical and adjuvant therapy, and the patient’s wishes are important.

## Data Availability

Not applicable.

## Abbreviations

LA: left atrium
TTE: transthoracic echocardiogram
TEE: transesophageal echocardiogram
PV: pulmonary vein
CECT: contrast enhanced computed tomography
MRI: magnetic resonance imaging
PML: posterior mitral leaflet
